# Assessment of factors affecting vaccine cold chain management practice in public health institutions in east Gojam zone of Amhara region

**DOI:** 10.1186/s12889-019-7786-x

**Published:** 2019-11-01

**Authors:** Hewan Adam Bogale, Abebe Feyissa Amhare, Alemtsehay Adam Bogale

**Affiliations:** 1 Clinton Health Access Initiative (CHAI), Pharmaceutical supply chain, regional coordinator on Child Survival, Bahir Dar, Ethiopia; 2 School of Public Health, Salale University, Fiche, Oromia Ethiopia; 3 Department of Pharmacy, Addis Ababa health bureau, Mesheualekia health center, Addis Ababa, Ethiopia

**Keywords:** Vaccine, Cold chain management, Immunization, Ethiopia

## Abstract

**Background:**

Maintaining quality of vaccines is one of the main challenges of immunization programs in Ethiopia. The objectiv**e** of this study is to assess the factor affecting vaccine cold chain management practice in immunization health institutions in East Gojam zone of Amhara region, Ethiopia.

**Method:**

An institutional based cross-sectional study was conducted from March to April 2017 in ten districts of East Gojam zone of Amhara Region. Descriptive statistics and Logistic regression analysis were carried out to identify factors related to the practice of cold chain management.

**Result:**

Among 60 health institutions, only 46(76.7%) had functional refrigerators. Twenty-one (35%) had a functional generator for backup service and 28(46.6%) had a car/motorbike for transportation of vaccines in case of refrigerator/power failure. Twenty-nine (48.3%) had known the correct vaccine storage temperature (2 °C – 8 °C) in the refrigerator and the results of this study revealed that only 23(38.3%) of respondents had sufficient knowledge about vaccine cold chain management. The finding of this study also revealed that 35(58.3%) had appropriate vaccine cold chain management practice and the rest 25(41.7%) had inappropriate practice. Logistic regression showed us the knowledge gap and profession were significantly associated with vaccine cold chain management practice at *P* < 0.05.

**Conclusion:**

This study indicates that there was a knowledge gap of health workers who are working on cold chain management. There is an urgent need to improve knowledge and practice on cold chain management through improved supervision and training at a different level of health care system.

## Background

Vaccination is the intervention used to prevent or eradicate childhood diseases. It is the most cost-effective health intervention. A set of practice guidelines for different service levels were created by the World Health Organization (WHO), which include vaccine monitoring, immunization techniques, cold chain management and reporting systems [1, 2]. Cold chain is system for storing and transporting vaccines in a potent state (within an acceptable temperature range) from the manufacturer to users [3]. Throughout the chain, primary health care providers must have adequate knowledge to manage the cold chain [4, 5]. The cold chain guidelines recommend the following: the vaccine storage should be maintained in the temperature range of 2–8 °C, the use of minimum/maximum thermometers, temperature charts, and the shake test [6–8]. However, these guidelines are often practically quite difficult to implement in field situations due to various factors like infrastructure problems and workload pressures [9–11].

Achieving high coverage rates is considered one of the parameters to measure the success of the immunization program. However, disease reduction which is the ultimate goal of the Expanded Program on Immunizations (EPI) depends mainly on the provision of potent vaccines to the children. Vaccine potency is ensured, among other things, by maintaining a functional cold chain system at all levels [12, 13].

The cold chain system is assumed to be at greatest risk, particularly in tropical countries where a power supply is unreliable and facilities for its maintenance are not well developed. In these areas, it is common to observe 30–50% of the refrigerators and freezers being out of order [14, 15]. Even a study done in the United States indicated that there is a lack of refrigerator temperatures that fell within the acceptable range which means 63% fell below minimum, 59% fell above maximum, and 93% fell either above or below or both [16].

In 1980s Immunization Supply Chain and Logistics (ISCL) systems supported acceptable vaccination coverage by using coping mechanisms to overcome problems in vaccine distribution, storage and management. As immunization programs grow, supply systems have coped with increased volume and complexity in several ways [17].

Cold chain management weaknesses are often observed during transportation and storage of the vaccines. Some factors contributing to weaknesses of the cold chain are delays during transportation, quality of refrigerators, a method of storage, too long storage at the health unit, improper use of refrigerators, power interruption, equipment breakage, and lack of trained personnel capable of managing the cold chain [5, 13, 18–21].

Cold chain system consists of a series of storage and transport links, all designed to keep vaccines within an acceptable temperature range until it reaches the users. The cold chain remains a highly vulnerable point for national immunization programs in developing countries especially those with tropical climates [1, 22, 23].

In Ethiopia, there is a real problem of vaccines losing their potency during storage at these centers even if they were potent on arrival. In the study area of the Amhara region, there are high immunization coverage rates, but there are still a number of reported outbreaks from different zones of the region. The aim of this study is to assess the factors affecting management and quality of the cold chain in immunization health institutions in East Gojam zone. Ethical clearance was obtained from the Research and Ethical Committee of Bahir Dar University, Ethiopia.

## Methods

The institution-based cross-sectional study design was conducted from March to April 2017 in East Gojam zone of Amhara region. East Gojam is one of the zone administrations of Amhara region. It has 18 districts and two town administrations. It was selected for this study because most of the districts are known for the frequent outbreak of vaccine-preventable diseases and frequent breakage of the cold chain.

The sample size was estimated using a single population proportion formula for a cross-sectional study. It was calculated by assuming 50% prevalence, marginal error (d) 0.05, and with 95% CI. Finally, the correction factor for the finite population was applied and the final sample was 60 health institutions.

Ten study districts were selected from 18 districts and two town administrations by simple random sampling. From the selected districts, 10 were districts health offices, 30 health centers and 20 health posts which have refrigerators for vaccine storage and the cold chain storekeeper were interviewed at district level, the under-five focal person (responsible person at immunization ward) was interviewed at the health center, and the health extension workers (community health workers) were interviewed at health post level until we reached final sample size.

The data was collected by using a structured questionnaire prepared for this study as shown in Additional file 1, which consists of four main categories namely socio-demographic variables, cold room infrastructure status, cold chain management knowledge which contain 7 items, and cold chain management practice variables which contain 13 items. The level of knowledge computed by considering the normal distribution of the data, a mean value 5 or above (≥75%) was considered as an appropriate cut-off point to classify knowledge as sufficient or insufficient. The level of practice was computed by considering the normal distribution of the data, a mean value 10 or above (≥75%) was considered as an appropriate cut-off point to classify cold chain practice as proper or improper practice [24].

English version of the questionnaire was translated into Amharic and back to English to check its conceptual equivalence. The Amharic version of the questionnaire was pre-tested in a non-sample health facility to assess for its clarity and correction were made accordingly.

The collected data were re-translated to English, entered into Epi Data software version-3.1 and transferred to SPSS software version-24, and analyzed according to the different variables. Binary and multivariate logistic regression analysis was performed to explore independent variables that are predictors of vaccine cold chain management practice were considered as significant when *P <* 0.05.

## Results

### General characteristics of health facilities

All 60 health facilities were participated (overall response rate was 100%). Among a total of 60 health facilities, 46(76.7%) had functional refrigerators, 21(35%) had a functional generator for backup service, and 28(46.6%) had a car/motorbike for transportation of vaccines in case of refrigerator/power failure. Fuel was available for only 43% generators, 57.1% car/motorbikes and 21.7% refrigerators. From the 60 health facilities with refrigerators, 63.3% had trained personnel and 20% had spare parts for minor maintenance.

### General characteristics of vaccine managers in the study health institutions

Among the vaccine providers included in the study, 34(56.7%) were females, 31 (51.6%) were nurses, 10(16.7%) were pharmacy personnel’s and 19 (31.7%) were health extension workers and 35 (58.3%) of the respondents were diploma graduates. Among these health care providers, 63.3% of the respondents had 2–5 years of total experience. The minimum and maximum years of service were 6 months and 12 years respectively. Out of the total respondents, 38 (63.3%) had received EPI training.

### Vaccine cold room storage status

Forty-nine (86.7%) of the health facilities hadn’t separate vaccine storage room and only 8 (13.3%) of them had a separate room for vaccine storage. About 15(25%) had sufficient storage capacity, 21(35%) were cleaned, 43(71.7%) had dry floors, 44(73.3%) were free of a roof leak and all storage area had secured windows and doors as shown in Table 1.

**Table 1:** Health worker reported status of vaccine cold room in health institutions

Characteristics	Frequency (*n* = 60)	Percent (%)
Cleanness of cold room		
Yes	21	35
No	39	65
Cold room adequate storage capacity		
Yes	15	25
No	45	75
Separate room for vaccines		
Yes	8	13.3
No	49	86.7
Roof free of leaks		
Yes	44	73.3
No	16	26.7
Windows and external room security		
Yes	60	100
No	0	0
Dryness of floor and in reasonable level		
Yes	43	71.7
No	17	28.3

### Knowledge of health care workers on vaccine cold chain management

Fifty-eight (96.7%) of the respondents knew that vaccines are heat sensitive drugs and half of the health care workers knew that freezing is harmful to vaccine storage, although only 21(35%) of storekeepers knew the correct placing of the thermometer. More than half (58%) of respondents had correctly interpreted the vaccine vial monitor (VVM) reading of each vaccine. Also, about 23(38.3%) of respondents had sufficient knowledge about vaccine cold chain management as shown in Table 2.

**Table 2:** Health workers response to questions around vaccine cold chain management

Characteristics	Frequency n = 60	percent
Knowing which vaccine are heat sensitive		
Yes	58	96.7
No	2	3.3
Knowing correct storage temp.(2-8 °C)		
Yes	29	48.3
No	31	51.7
Store keeper knows freezing is harmful		
Yes	30	50
No	30	50
Correct placing of thermometer		
Yes	21	35
No	39	65
Correctly interpreted temperature reading		
Yes	41	68.3
No	19	31.7
Correctly Conducting shake test		
Yes	32	53.3
No	28	46.7
Correctly interpreting VVM reading		
Yes	35	58.3
No	25	41.7
Over all knowledge		
Sufficient knowledge	23 37	38.3
Insufficient Knowledge		61.7

### Vaccine cold chain management practice

**Fig. 1:**
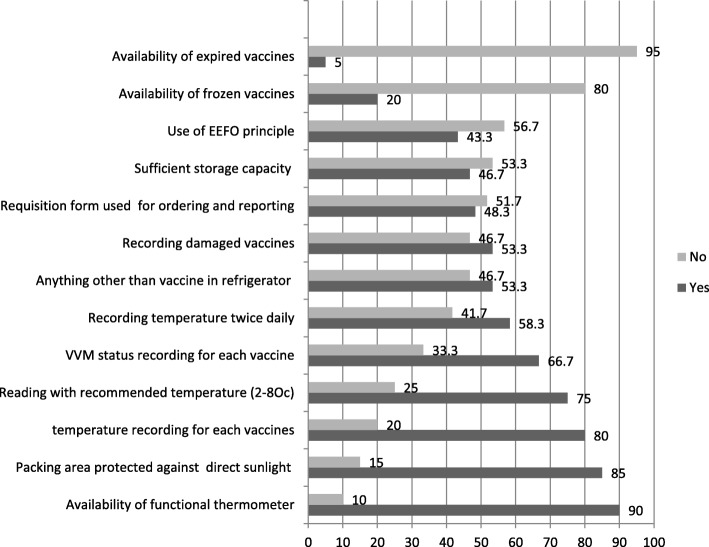
Vaccine cold chain management practice of health care workers

Regarding vaccine cold chain management practice, based on the recommended vaccine storage temperature range (+ 2 °C to + 8 °C), 25% of health facilities had a functional thermometer. From 60 health institutions, most of them (90%) had a functional thermometer, 66.6% of them had vaccines in good VVM status, and 46.7% of them had sufficient vaccine storage capacity. More than half of the refrigerators of the 60 health institutions used to store other material, and only 43.3% apply First Expiry First Out (FEFO) principle (the method of vaccine management involves issuing products with the earliest expiry date first, regardless of the order in which they are received) to reduce vaccine wastage. About 58.3% of health institutions had recorded cold room temperature twice daily continuously, and 80% of the health institutions had vaccine transaction recording practice as shown in Fig. 1.

### Factors associated with vaccine cold chain management practice

The finding of this study revealed that 35(58.3%) had cold chain management that conforms to the required standards and the rest 25(41.7%) has improper practice. Among the factors, only professional qualification and overall knowledge on vaccine cold chain management had a statistically significant association with the practice of health workers on cold chain management. Professional qualification, years of experience, overall knowledge of respondents on cold chain management, sufficient storage area was included in the regression model. The adjusted model indicated that respondents who had sufficient knowledge were 2 times more likely to have proper cold chain management practice as compared with those who had not. Nurses were about 17 times more likely to have proper practice on cold chain management compared to Health Extension Workers (AOR = 17.13 (2.58–11.34)). In addition, health workers who had sufficient overall knowledge were about 2 times more likely to have appropriate practice on cold chain management (AOR = 2.14 (0.03–1.72) as shown in Table 3.

**Table 3:** Bivariate and multivariate analysis of providers’ Cold Chain Management practice

Variable	Over all practice		Odds Ratio		*P*-value
	Inappropriate N (%)	Appropriate N (%)	COR (95%CI)	OR (95%CI)	
Overall knowledge					
Sufficient knowledge	3 (13.0)	20 (87.0)	15.4 (4.3–55.9)	2.14 (0.3–1.72)	0.018
Insufficient knowledge	22 (59.5)	15 (40.5)	1.00	1.00	
Profession					
Nurse	14 (45.2)	17 (54.8)	0.104 (.03–0.4)	17 (2.58–11.34)	0.003
Pharmacy	8 (80)	2 (20)	0.04 (.04–.40)	0.032 (2.35–7.56)	0.04
Health Extension	3 (15.8)	16 (84.2)	1.00	1.00	

## Discussion

This study identified the gaps related to cold chain system management. There were gaps in following the cold chain management principles such as use of EEFO principle, recording damaged vaccine, recording temperature twice daily, and recording VVM status for each vaccine. Also, there was a gap in using requisition form for reporting and ordering vaccine. Based on the descriptive analysis, the majority of the health facilities did not have a separate room for vaccine storage. Their storage capacities were insufficient to accommodate all vaccines and its consumable supplies, even though all storage area had secured windows and doors. Infrastructures were not fulfilled in room to handle the vaccine properly. This inappropriate handling of the vaccine has a negative effect on its potency. This study identified only 35 health facilities (58.3%) which had proper vaccine cold chain management practice. This finding is very low when compare with study done in Saudi which showed that handling and usage of vaccines during immunization sessions at government health facilities was more than 90% appropriate [25]. The possible reasons could be the educational and income difference of the countries.

The availability of vaccine storage equipment was found to be unacceptable in East Gojam zone of Amhara Region. These are enough to attend the desired level of vaccination coverage if appropriately utilized. However, the availability of the power sources and an alternative source were not sufficient. The number of health facilities using electricity as their primary power source predominates but the major challenge of this type of power supply was an irregularity in the power supply. Availability of necessary spare parts and conducting cold room maintenance were very low.

In this study, 76.7% of health facilities had functional refrigerators, which is much better as compared to the study conducted in central Ethiopia which reported that only 19% had functional refrigerators [10]. The possible reason may be an unequal distribution of health facilities or improper handling.

According to the recommended vaccine storage temperature range (+ 2 °C to + 8 °C), 25% of health facilities had a functional thermometer in current study which is consistent with a study done in central Ethiopia, [18] and lower as compared with the study done in Cameroon which had 32.7% functioning thermometers, [9]. Even if both are developing counties, the possible reason may be due to unequal distribution of health facilities and inappropriate handling of thermometer.

The cold chain monitoring status in the East Gojam Zone of Amhara Region was inadequate to ensure proper vaccine storage. In this study, health personnel knowledge and practices being inadequate to support maintenance of effective cold chain standards, which means there were cold chain management knowledge and practice gaps as per effective vaccine management standards. The results of this study revealed that only 38.3% of respondents had sufficient knowledge about vaccine cold chain management which is much lower than a study done on cold chain status for immunization in central Ethiopia in which 56% of health workers had satisfactory knowledge on cold chain management [18]. Potential factors associated which likely to be responsible for this low level of cold chain management practices include insufficient knowledge or training, and insufficient support.

Since the study was conducted in 60 health institutions due to time and resource limitation, there was a reduced statistical power, caution is needed in generalizing these results. The cross-sectional design investigates prevalence and associations rather than causality. Thus, future research needs to replicate these findings using a large-scale sample size to generalize and using qualitative and quantitative study design producer at different levels of the health system.

## Conclusions

In general, the findings of this study indicate that there are important knowledge gaps for health workers in managing the cold chain management. Most cold rooms have insufficient storage capacity and incomplete equipment for adequate cold chain maintenance. Vaccine cold chain management was significantly associated with the overall cold chain management knowledge and profession of a health care worker in health institutions. To maintain a safe and effective cold chain further efforts are needed both in education and training with improving health worker knowledge and with improving both infrastructure capacity and appropriate equipment. In addition, the regional health bureau and district health office should work on different capacity building activities to health professionals in order to address the knowledge gap at a different level of the health institution.

## Supplementary information

**Additional file 1.** Questionnaire of this study.

## Data Availability

The datasets collected and analyzed for the current study is available from the corresponding author and can be obtained on a reasonable request.

## Abbreviations

AOR: Adjusted odds ratio
EPI: Expanded program on immunizations
FEFO: First expiry first out
ISCL: Immunization supply chain and logistics
VVM: Vaccine vial monitor
WHO: World Health Organization
