# Supplementary material for: Assessment of factors affecting vaccine cold chain management practice in public health institutions in east Gojam zone of Amhara region
Source: BMC Public Health. 2019 Nov 1;19:1433. doi: 10.1186/s12889-019-7786-x (PMC6824010; doi:10.1186/s12889-019-7786-x)
Supplement: Supplementary file 1 — Additional file 1. Questionnaire of this study. [file 12889_2019_7786_MOESM1_ESM.docx]

# English questionnaire

Questionnaire **for the assessment of factors associating with cold chain management of vaccines in East Gojjam zone of Ethiopia**

1. **Socio-demographic characteristics of the respondents**

| No | Characteristics |  |
| --- | --- | --- |
| 1.1. | Gender | 1. Male [ ] |
|  |  | 1. Female [ ] |
|  |  |  |
| 1.2. | Level of education |  |
|  |  | 1. Bachelor degree [ ] |
|  |  | 1. Diploma [ ] |
|  |  | 1. Certificate [ ] |
|  |  |  |
| 1.3 | Position |  |
|  |  | 1. Nurse [ ] |
|  |  | 1. Midwifery [ ] |
|  |  | 1. Other [ ] |
|  |  |  |
| 1.4 | Years of service | 1. <2 years [ ] |
|  |  | 1. 2-5 years [ ] |
|  |  | 1. 6-10 years [ ] |
|  |  | 1. >10 years [ ] |
|  |  |  |
| 1.5. | Have you ever taken EPI training? | 1. Yes [ ] |
|  |  | 1. No [ ] |
|  |  |  |
| 1.6 | Level of health institution | 1. Hospital [ ] |
|  |  | 1. Health center [ ] |
|  |  | 1. Health post [ ] |
|  |  |  |
| 1.7 | Availability of guide line in the facility | 1. Yes [ ] |
|  |  | 1. No [ ] |
|  |  |  |

1. **Availability of cold chain equipment in the health facilities**

| No | Parameters | Available | Not available |
| --- | --- | --- | --- |
| 2.1 | Functional backup generator in the facility |  |  |
| 2.2 | Functional car/motorbike to use in case of refrigerator failure |  |  |
| 2.3 | Trained person for minor maintenance |  |  |
| 2.4 | Spare part for minor maintenance |  |  |
| 2.5 | Permanently assigned personnel for cold chain follow up |  |  |
| 2.6 | Fuel for generator |  |  |
| 2.7 | Fuel for refrigerator |  |  |
| 2.8 | Availability of fuel for motor/car |  |  |

1. **Exterior of cold chain equipment**

| **No** | **Parameters** | **Yes** | **No** |
| --- | --- | --- | --- |
| 3.1 | Is the cold room clean, free of dirt? |  |  |
| 3.2 | Is the floor dry in acceptable level? |  |  |
| 3.3 | Is the roof free of leak? |  |  |
| 3.4 | Is vaccine separate storage area? |  |  |
| 3.5 | Is the windows and external room security maintained for vaccine? |  |  |
| 3.6 | Has the cold room sufficient area to store all vaccines and its consumables? |  |  |

1. **Cold chain management practice**

| **No** | **Parameters** | **Yes** | **No** |
| --- | --- | --- | --- |
| 4.1 | Anything other than the vaccine stored in refrigerator? |  |  |
| 4.2 | Has the vaccine room sufficient storage capacity? |  |  |
| 4.3 | Is vaccines properly arranged in refrigerator? |  |  |
| 4.4 | Is the vaccine management following FEFO principle? |  |  |
| 4.5 | Are there expired vaccines present in refrigerator? |  |  |
| 4.6 | Is there frozen vaccines present in refrigerator? |  |  |
| 4.7 | Is refrigerator temperature maintained between 2-8^0^C? |  |  |
| 4.8 | Is there functional thermometer for refrigerator? |  |  |
| 4.9 | Is the refrigerator T^0^ recorded twice daily? |  |  |
| 4.10 | Is the vaccine packing area protected from direct sun light? |  |  |
| 4.11 | Are all vaccine transactions recorded? |  |  |
| 4.12 | Is VVM status of vaccine recorded for each vaccine? |  |  |
| 4.13 | Is there expired vaccine in refrigerator? |  |  |
| 4.14 | Is there vaccine LMIS reporting and requisition form done in the last month? |  |  |

1. **Knowledge and awareness regarding cold chain**

| **No** | **Characteristics** | **know** | **Not know** |
| --- | --- | --- | --- |
| 5.1 | Do you know that vaccines are heat sensitive? |  |  |
| 5.2 | Do you know that freezing is harmful for vaccines? |  |  |
| 5.3 | Do you know the correct placing practice of thermometer? |  |  |
| 5.4 | Do you know the correct demonstration of temperature reading? |  |  |
| 5.5 | Do you know the correct temperature for vaccine storage (2-8^0^C)? |  |  |
| 5.6 | Do you know the correct interpretation of VVM? |  |  |
| 5.7 | Do you know the correctly interpreted shake test? |  |  |
